# Facile synthesis of PEI-based crystalline templated mesoporous silica with molecular chirality for improved oral delivery of the poorly water-soluble drug

**DOI:** 10.1080/10717544.2021.1912212

**Published:** 2021-05-07

**Authors:** Wei Xin, Yumei Wang, Yan Bian, Jiahui Lin, Wenhao Weng, Xinyi Zhao, Kaijun Gou, Xianmou Guo, Heran Li

**Affiliations:** a School of Pharmacy, China Medical University, Shenyang, China; b The First Affiliated Hospital of China Medical University, Shenyang, China; c School of Pharmacy, Shenyang Pharmaceutical University, Shenyang, China

**Keywords:** Biomimetic synthesis, chiral mesoporous silica, drug delivery, oral bioavailability, nimodipine

## Abstract

The aim of this study was to build up a novel chiral mesoporous silica called PEIs@TA-CMS through a facile biomimetic strategy and to explore its potential to serve as a drug carrier for improving the delivery efficiency of poorly water-soluble drug. PEIs@TA-CMS was synthesized by using a chiral crystalline complex associated of tartaric acid and polyethyleneimine (PEIs) as templates, scaffolds and catalysts. The structural features including morphology, size, pore structure and texture properties were systematacially studied. The results showed that PEIs@TA-CMS was monodispersed spherical nanoparticles in a uniformed diameter of 120–130 nm with well-developed pore structure (S_BET_: 1009.94 m^2^/g, pore size <2.21 nm). Then PEIs@TA-CMS was employed as nimodipine (NMP) carrier and compared with the drug carry ability of MCM41. After drug loading, NMP was effectively transformed from the crystalline state to an amorphous state due to the space confinement in mesopores. As expected, PEIs@TA-CMS had superiority in both drug loading and drug release compared to MCM41. It could incorporate NMP with high efficiency, and the dissolution-promoting effect of PEIs@TA-CMS was more obvious because of the unique interconnected curved pore channels. Meanwhile, PEIs@TA-CMS could significantly improve the oral adsorption of NMP to a satisfactory level, which showed approximately 3.26-fold higher in bioavailability, and could effectively prolong the survival time of mice on cerebral anoxia from 10.98 to 17.33 min.

## 1. Introduction

In the area of pharmaceutical industry, one of the particularly widespread challenges to the development of highly potent pharmaceutics is the poorly water-soluble of a large number of drugs (Perez et al., 2017; Chen et al., 2018; Zhang et al.,^2018^). Although several factors may be responsible for the low bioavailability of drug, the main reason is the low solubility and the resulting incomplete drug dissolution (Karki, et al., 2018). Particularly for Biopharmaceutical Classification System class II (BCS II) drugs with low soluble and high permeable, a slight increase in solubility may contribute to a significant improvement in the bioavailability. Furthermore, the practically insoluble of therapeutic drugs has been associated with a series of clinical obstacles, such as insufficient bioavailability, poor absorption, inability in dose–response proportionality, and slow clinical outcomes; especially in oral delivery which is still the most preferable administration pathway with the best patient compliance (Elshaer, et al., 2011). However, nearly 40% of marketed drugs suffer from poor solubility; for example, nimodipine (NMP), which is a 1,4-dihydropyridine derivative belonging to the second generation calcium channel blockers. NMP is commonly used for the prophylaxis and treatment of resultant ischemia and cerebral vasospasm induced by the subarachnoid hemorrhage (Pickard et al., 1989; Ahmedet al., 2000; Serajuddin, 2007). Due to the highly lipophilic nature, it is allowed to cross through the blood-brain barrier, thus exhibiting a stronger effect on cerebral arteries than on arteries elsewhere in the body (Pickard et al., 1989; Knapik-Kowalczuk et al., 2018; Zhang et al., 2018). However, as a BCSII drug, the poorly solubility of NMP always led to extremely low oral adsorption. As verified by researchers, a strong first-pass effect in the liver further results in unsatisfactory oral bioavailability (between 0% and 13%) (Zhang et al., 2018).

Several solubility-enhancement strategies (e.g. salt formations (Serajuddin, 2007; Park et al., 2019), micronizations (Martin & Cocero, 2008), microsizing or nanosizing (Junyaprasert & Morakul, 2015; Ramachandraiah et al., 2018; Ren et al., 2019), surfactants (Morgado et al., 2020) or polymeric micelles (Chiappetta & Sosnik, 2007) and solid dispersions (Phuong et al., 2019)) and effective preparation formulations (e.g. lipid-based formulations (David, 2007), hydrogels (Benoit et al., 2006), liposomes (Jung et al., 2020), cyclodextrins (Mrówczyński et al., 2018) and vesicles (Mazzotta et al., 2018)) have been employed to overcome these obstacles. There are still several limitations for the traditional pharmaceutically acceptable carrier materials, for example, the high cost in synthesis, the poor thermal/chemical stability, the low and erratic drug loading efficiency, and the lack of biological inertness, etc. (Singh et al., 2011.). On account of the demand for more effective stabilizer in nanoparticle formulations, drug delivery with the assistance of inorganic mesoporous silica materials are one of the most novel and important technologies for enhancing drug dissolution. Since a MCM41 based drug release systems was first reported in 2001, mesoporous silica nanoparticles (MSNs) as delivery vehicles have attracted growing attention owing to their advantageous structrural properties, such as highly regular mesoporous structure, large surface area and pore volume, ordered and tunable pore size, rich and diversified morphology, good biocompatibility, and ease of surface (including the inner pore system and/or the external surface) functionalization (Wang et al., 2015; Perez et al., 2017). Furthermore, compared with the traditional “soft” pharmaceutical drug carriers, one of the most prominent advantages of MSNs is the physiological inertness and mechanical stability, which can provide sufficient protect for their cargo while performing the delivery task *in vivo* (Slowing et al., 2007). These features make MSMs promising candidates for delivery various therapeutic agents (Li et al., 2019; Dement'Eva et al., 2020).

As far as we know, among many parameters that may play influential roles, the porous structure is of especial great importance and often characterizes MSNs. Common mesopore phases in silica with pore sizes between 2 and 5 nm are 2 D hexagonal p6m, 3 D cubic Ia3d and lamellar p2, such as MCM41, MCM48 and MCM50 (Perez et al., 2017; Wang et al., 2015). Among them, MCM41 exhibiting a long-range ordered pore structure is the most widely used type of silica-based materials for drug delivery, and are discovered to enhance the dissolution rate of a series of hydrophobic drugs, including ibuprofen, efavirenz, telmisartan, etc. (Wang et al., 2015; Jesus et al., 2016; Izquierdo-Barba et al., 2009; Horcajada et al., 2004; Wang et al., 2014). By using an amphiphilic triblock copolymer Pluronic P123 as a template, SBA15 with large mesopores (pore size: 5–30 nm) was obtained. Benefiting from the wide mesopores, drug could release from SBA15 faster compared to MCM41, and a different dynamic release performance was observed (Jesus et al., 2016; Izquierdo-Barba et al., 2009). Meanwhile, Li et al developed a drug delivery system involving MSNs with bimodal nanoporous, and the *in vitro* release study suggested two phases controlled release behavior including an immediate release from the enlarged nanopores and a second dissolution phase from the smaller mesoscopic channels (Li et al., 2016). Besides, the results reported by Hu et al. indicated that drug dissolved from SBA16 with an opened 3 D cage-like cubic pore structure exhibited a fast release rate (Hu et al., 2012). Li et al. also revealed that MSNs with disordered nanopores showed a quicker release behavior as a result of a shorter diffusion distance (Li et al., 2018). It seems that pore morphology is thought to greatly affect the drug loading and release behavior (Wang et al., 2015, Li et al., 2018). Among all the pore structural factors, chirality, which is defined as a geometric asymmetry through translation and rotation, is the fundamental issue in the relative fields of chemistry, physics, biology, and medicine (Che et al., 2004; Jin et al., 2006). In addition, it is expressed as one of the inherent attributes of the molecular and macromolecular components in organisms (Che et al., 2004). Naturally, applying chirality definition into MSNs, chiral mesoporous silica (CMS) has been proposed and have been of significant interest in a variety of fields. Notably, the bold attempt of applying CMSs as drug carriers in recent years has brought unlimited possibilities due to the beneficial property of chiral materials (Wu et al., 2021). Previous reports point out that CMS has several interesting features, such as chiral topological structure (in molecular level, supramolecular level or macroscopic level), interconnection between individual porous channels, high external surface area and potential chiral recognition functions (Wang et al., 2019; Gou et al., 2020a,b).

In views of the potential advantages, we designed a new kind of CMS namely PEIs@TA-CMS at neutral pH and ambient conditions, and further study its performance as a delivery platform for poorly water-soluble drug. In the synthesis process, a chiral crystalline complex was facile synthesized by grafting tartaric acid onto the long chains of linear PEIs through electrostatic interaction and was then acted as scaffolds, catalysts and templates to build up PEIs@TA-CMS. Along with the deposition of silica product, PEIs@TA-CMS with geometrically ordered chiral blocks on its surface was obtained. The obvious highlights and characteristics of as-synthesized PEIs@TA-CMS were as follows: (1) CMS was able to be facile synthesized at ambient conditions (ambient temperature, neutral pH and ambient pressure); (2) the synergistic effect from the linkage of carboxyl groups with amine groups could actively promote silica deposition; (3) The reactants PEIs, water and L-tartaric acid were all nontoxic, and can be associated to form chiral crystalline complex through very simple mixing process. The structural features of PEIs@TA-CMS were characterized by using Fourier transform infrared spectroscopy (FTIR), scanning electron microscope (SEM), transmission electron microscope (TEM), and nitrogen adsorption and desorption tests. In order to improve the drug dissolution and oral bioavailability, NMP was selected as the model drug and was incorporated into PEIs@TA-CMS according to the organic solvent drying method. FTIR, differential scanning calorimetry (DSC) and X-ray diffractometry (XRD) analysis were conducted to confirm the physicochemical properties of samples before and after drug loading. The drug loading amount and *in vitro* release behaviors were investigated to evaluate the host capacity and controlled release properties of PEIs@TA-CMS. Finally, *in vivo* pharmacokinetics study and *in vivo* pharmacodynamics study were performed to evaluate the potential of PEIs@TA-CMS on improving the oral adsorption of NMP.

## 2. Materials and methods

### 2.1. Chemicals and reagents

Tetraethoxysilane (TEOS) was obtained from Aladdin (Shanghai, China). Linear polyethyleneimines and L-tartaric acid (L-TA) was provided by Chengdu Xiya Chemical Technology Co., Ltd. (Chengdu, China). Nimodipine was obtained from Wuhan Fungzhilin Chemical Co., Ltd. (Wuhan, China). MCM41 (particle size, 200–300 nm; average pore diameter, 3–4 nm) was purchased from Suzhou Jiedi Nano Technology Co., Ltd., (Suzhou, China). Double deionized water was prepared by using ion exchange method. All other chemicals were of reagent grade and used without any further purification.

### 2.2. Synthesis of PEIs@TA

**Figure 1.**
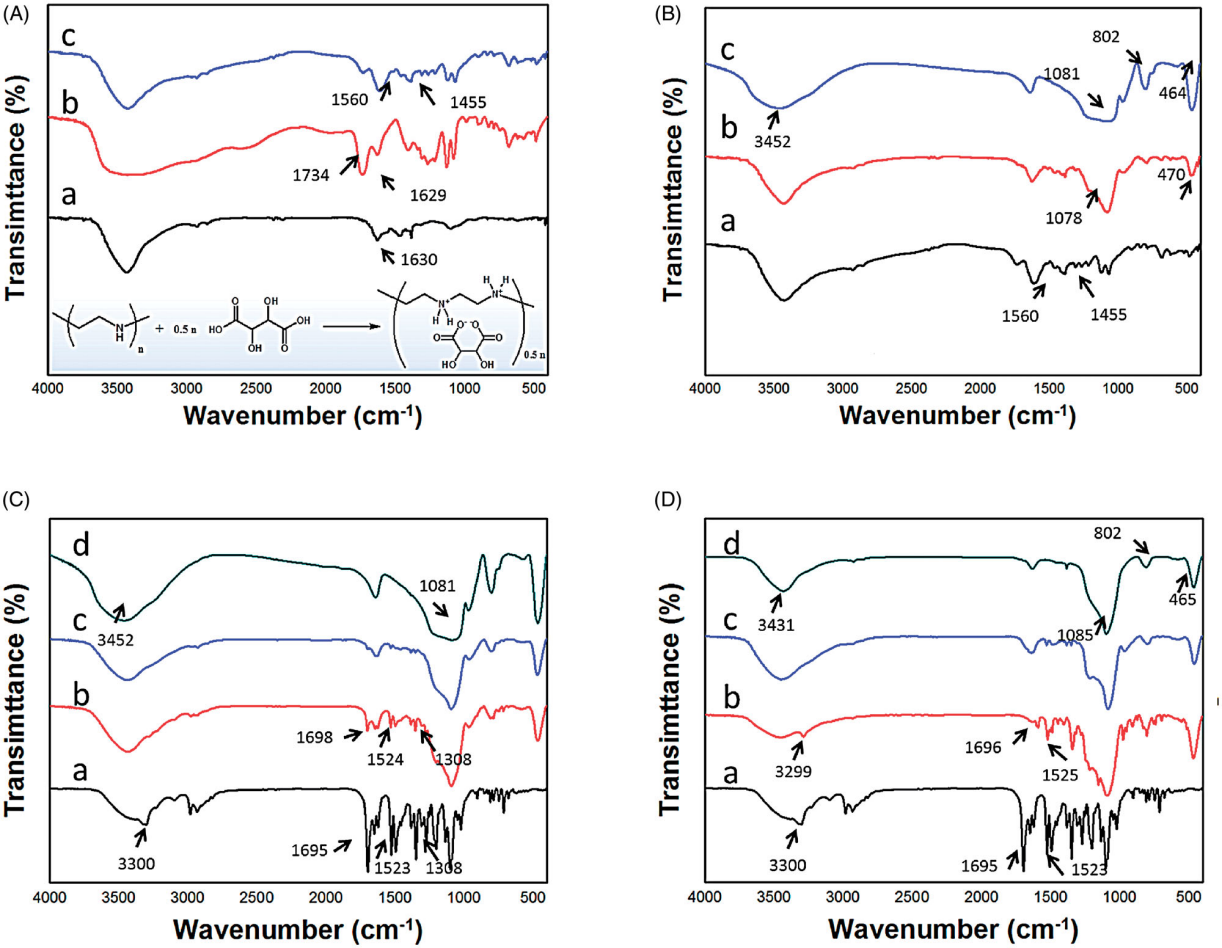
(A) FTIR spectra of (a) PEIs, (b) L-tartaric, (c) PEIs@TA, and the representation of the formation of PEIs@TA (in the embedded response equation); (B) FTIR spectra of (a) PEIs@TA, (b) PEIs@TA-CMS before calcine, and (c) PEIs@TA-CMS after calcine; (C) FTIR spectra of (a) NMP, (b) the physical mixture of NMP and PEIs@TA-CMS, (c) NMP/PEIs@TA-CMS, and (d) PEIs@TA-CMS; (D) FTIR spectra of (a) NMP, (b) the physical mixture of NMP and MCM41, (c) NMP/MCM41, and (d) MCM41.

As described in Figure 1(A), a chiral nanocrystalline aggregates registered as PEIs@TA was prepared by using a chiral crystalline complex associated of chiral tartaric acid and polyethyleneimine (Matsukizono & Jin, 2012.). In a typical run, 0.316 g PEIs was dispersed into 1 mL deionized water under stirring followed by the addition of 0.3 g L-tartaric acid at 60 °C. 4 h later, the solution was cooled down in an ice-water bath, and left for 20 min for aggregation. The resulting white solid species named PEIs@TA was recovered by suction filtration and washed multiple times with deionized water.

### 2.3. Synthesis of PEIs@TA-CMS

For the first time, a new type of chiral mesoporous silica named as PEIs@TA-CMS was synthesized by using the chiral crystalline complex PEIs@TA formed from chiral tartaric acid and PEIs as catalysts, scaffolds and templates. In a typical run, the obtained PEIs@TA was dispersed into 30 mL deionized water under vigorous stirring. After that, 6 mL TEOS was immediately added to the system, stirred continuously for 24 h, and statically treated for another 24 h. The product was collected by centrifugation, washed by ethanol and water for several times, and dried thoroughly. Finally, the dried sample was exposed to thermal treatment at 550 °C for 6 h with a slow heating rate to get PEIs@TA-CMS.

### 2.4. Characterization of carriers

To confirm the successful synthesis of PEIs@TA and PEIs@TA-CMS, FTIR study was carried out by Spectrum 1000 spectrometer (Perkin Elmer, USA) over the spectral region of 400–4000 cm^−1^. KBr pellet method was used in this study; samples were carefully mixed with dried KBr and were compressed into tablets before analyzing.

The morphology and mesoscopic structure of PEIs@TA-CMS and MCM41 was observed using a TEM instrument (Tecnai G2-F30, FEI, The Netherlands) and a SEM instrument (JSM-6510A, JEOL, Japan). The TEM samples were prepared by dispersing the sample in ethanol through sonication. Subsequently, one drop of the solution was deposited on a carbon-copper grid and the sample was dried for 10 min under an infrared lamp. Before the SEM examination, samples were attached on the double side adhesive carbon tape, and then gold-coated under vacuum condition. Meanwhile, the particle size distributions were accounted according to the SEM images (calculated from 200 nanoparticles) The size distribution was further studied using a Zeta-Potential/Particle Sizer (3000 HAS, UK) and the value was the average of three consecutive measurements.

Nitrogen desorption/adsorption tests were performed on a V-Sorb 2800 P adsorption analyzer (Gold APP, China) to give information on the texture properties and pore structure of PEIs@TA-CMS and MCM41. Prior to the testing, the sample was degassed at 120 °C for at least 6 h. The specific surface area (S_BET_) of the sample was calculated on the base of the Brunauer–Emmett–Teller (BET) method, while the pore volume and pore size distribution were acquired by using the Barrett–Joyner–Halenda (BJH) method derived from the desorption branches of the isotherms.

### 2.5. Drug loading procedure

The poorly water-soluble drug nimodipine (classified as BCS II) was selected as a model drug, and was loaded into PEIs@TA-CMS according to solvent deposition method, which had been proved to effective method for the preparation of drug loaded samples. This method involves two stages, including an initial adsorption equilibrium step and a solvent evaporation step. The high-concentration NMP ethanol solution was prepared by dissolving 20 mg of NMP into 2 mL ethanol. A series of NMP loaded PEIs@TA-CMS samples (NMP/PEIs@TA-CMSs) was achieved by adding PEIs@TA-CMS to the ethanol solution of NMP at the drug: carrier ratio of 1:1, 1:2 and 1:3 (w/w), respectively. Then the system was sealed and stirred for 24 h to assure the maximum drug loading and dried under vacuum to remove the organic solvent. Afterwards, NMP/PEIs@TA-CMSs was washed using a small volume of ethanol (2 mL) to remove the drug adsorbed on the surface of carrier, and thoroughly dried subsequently. Samples were named as NMP/PEIs@TA-CMS (1:1), NMP/PEIs@TA-CMS (1:2) and NMP/PEIs@TA-CMS, respectively. NMP/MCM41 sample was acquired by the same method at the drug/carrier ratio of 1:3 (w/w). Meanwhile, physical mixtures of NMP with PEIs@TA-CMS and MCM41 were prepared through a simple mixing process of drug and carriers (1:3, w/w) and regarded as references.

To measure the drug loading content, an accurately weighed quantity of NMP/PEIs@TA-CMSs was dispersed in 100 mL ethanol, and extracted completely under ultrasound. After passing through the 0.22 μm polytetrafluoroethylene (PTFE) membrane filter, the drug concentration was determined by ultraviolet (UV) spectroscopy (UV-1750, Shimadzu, Japan) at a wavelength of 238 nm.

### 2.6. Characteristics of NMP loaded carriers

To analyze the interactions between drug and carrier, FTIR study of NMP, PEIs@TA-CMS, MCM41, physical mixtures, NMP/PEIs@TA-CMS and NMP/MCM41 was carried out by Spectrum 1000 spectrometer (Perkin Elmer, USA) over the spectral region of 400–4000 cm^−1^. Moreover, XRD measurement was carried out on an X-ray diffractometer (X'pert PRO, PANalytical, Netherlands) to analyze the typical changes in physical state of samples before and after NMP loading. The data was scanned at the diffraction angle range of 5° to 40° (2*θ*) with a scanning speed of 0.05°. DSC test of NMP, carriers, physical mixtures, and drug loaded samples was conducted on a thermal analyzer (Q1000, TA Instrument, USA) by heating samples from 25 to 200 °C under a N_2_ flow at the step of 10 °C/min.

### 2.7. *In vitro* release of NMP

The *in vitro* release behavior of samples was assessed by a USP II paddle method on a dissolution apparatus (ZRS-8G, Huanghai Medicament Test Instrument, China). Prewarmed medium of 0.5% (w/v) sodium dodecyl sulfate (SDS) solution (250 mL, 37 °C) was added to the dissolution cup with a stirrer rotation speed of 50 rpm. 10 mg NMP, NMP/MCM41 and NMP/PEIs@TA-CMSs samples containing 10 mg of NMP were suspended in the release medium. 5 mL of aliquots were collected and replaced by an equal volume of fresh isothermal supplement at predetermined time intervals, After filtering through a 0.22 μm membrane filter, the drug release amounts were tested by UV spectroscopy (UV-1750, Shimadzu, Japan) at 238 nm. All *in vitro* release studies were repeated three times.

### 2.8. *In vivo* pharmacokinetics

Animal experiments were approved by the Institutional Animal Care and Use Committee at China Medical University. All rats were maintained complied with guidelines for the Care and Use of Laboratory.

Twelve male Sprague-Dawley rats (SD rats, 180–220 g body weight) were randomly divided into three groups (*n* = 4): NMP group, NMP/PEIs@TA-CMS group and NMP/MCM41 group. Prior to the experiment, animals were fasted overnight with free access to water. The animals were orally administered the water suspensions of NMP, NMP/PEIs@TA-CMS and NMP/MCM4 with a drug dose of 2.5 mg. At appropriate sampling times, aliquot blood sample (about 0.6 mL) was withdrawn via the retro-orbital venous sinus, centrifuged to separate the plasma, and stored at −20 °C.

In the pretreatment procedure, 20 μL of internal standard solution (nitrendipine, 20 μg/mL) was added into 200 μL plasma sample and vortexed for 1 min. Afterwards, 100 μL NaOH (0.01 mol/L) was uniformly mixed with the system and vortexed for 1 min. The drug was extracted by 2.5 mL diethyl ether, vortexed for 5 min, and centrifugated for 12 min to separate the organic layer (2 mL). The organic layer (2 mL) was then transferred to a new tube and evaporated to dryness under a gentle stream of N_2_. After reconstituting with 100 μL mobile phase, the 20 μL sample was analyzed by high performance liquid chromatography (HPLC) method. The separation procedure was performed on a Kromasil ODS-2C_18_ column (Thermo ODS-2 Hypersil, 250 mm × 4.6 mm, 5 mm, UK) equipped with a JanuSep C_18_ pre-column (Liaoning, China), and the column temperature was kept at 25 °C. The UV detector was set at 240 nm. The mixture of methanol, acetonitrile and water (3:4:3, v/v/v) was selected as the optimized mobile phase with a flow rate of 1 mL/min. The NMP content was calculated according to internal standard method, and the main pharmacokinetic parameters were obtained using the software of DAS 2.0.

### 2.9. *In vivo* pharmacodynamics

The pharmacodynamics study was performed to further evaluating the effect of PEIs@TA-CMS on improving the oral adsorption of NMP. 36 SPF-grade Kunming mice (18–22 g body weight) were assigned into six groups, randomly (*n* = 6): Normal saline group, NMP group, NMP/PEIs@TA-CMS (low dose) group, NMP/PEIs@TA-CMS (middle dose) group, NMP/PEIs@TA-CMS (high dose) group and NMP/MCM41 group. Before dosing, animals were fasted overnight with free access to water. Animals in the Normal Saline group were received oral treatment of 0.5 mL normal saline, and were considered as negative control. While for NMP group which severed as positive control, 0.5 mg pure NMP was suspended in 0.5 mL normal saline and was imposed to animals. Normal saline suspensions of NMP/PEIs@TA-CMS and NMP/MCM41 containing 0.5 mg NMP were also oral administrated to mice. Meanwhile, half and twice of the dose in NMP/PEIs@TA-CMS (middle dose) group, which were respectively in doses equivalent to 0.25 mg and 1 mg NMP, were given to mice to further study the influence of dose. To make sure that NMP had been circulated in the body to exert an effect on cerebral vasospasm, the cerebral anoxia of mice was established by intraperitoneal injection of NaNO_2_ (0.4 mL, 0.2%, w/v) 90 min post oral administration. Then the clinic manifestation and survival time of mice was recorded to study the protective effect of NMP formulations on mice cerebral anoxia.

### 2.10. Statistical analysis

All data were presented as the mean ± SD and were analyzed using one-way ANOVA. Student’s *t*-test was conducted to determine identify levels of significance between the groups with statistically significance considered as *p* < .05.

## 3. Results and discussion

### 3.1. Formation mechanism of PEIs@TA-CMS

In the present study, a novel CMS constructing program was established by using soft nanocrystalline aggregates self-organized from a chiral crystalline complex by association of PEIs with tartaric acid. Upon the addition of tartaric acid, the negatively charged –COOH groups electrostatically interacted with the –NH– sites from the aqueous solution of PEIs. Due to the small steric hindrance, two carboxyl groups of L-tartaric acid functioned with amino group of PEIs, and the chiral conformation in tartaric acid was endowed. Chiral soft nanocrystalline aggregates (denoted as PEIs@TA) could be obtained by cooling the mixture to induce crystallization, and then functioned as templates to direct the structure of PEIs@TA-CMS. It should be mentioned that, the synergistic effect from the association of carboxyl groups with amine groups was reported to facilitate the silica condensation (Matsukizono & Jin, 2012). After the addition of TEOS, PEIs@TA also acted as catalysts and scaffolds, and effectively promoted the hydrolysis and deposition process of silica precursors. Employing PEIs@TA as templates, scaffolds and catalysts, we performed the silicification under neutral pH and ambient conditions, which all in coincide with the principle of biomimetic synthesis (Jin & Yuan, 2005; Wang et al., 2012). Meanwhile, by introducing the chirality into the long chains of PEIs, chiral topology structure was imprinted along with silica deposition.

The successful synthesis of PEIs@TA and PEIs@TA-CMS were verified by FTIR. As shown in Figure 1(A), for PEIs, absorptions assigned to C–H stretching vibrations at 2800–2900 cm^−1^ and the bending vibration of N-H at 1630.4 cm^−1^ could be noticed (Li et al., 2014). The FTIR spectra of tartaric acid showed carboxylic acid characteristic absorption, which were stretching vibrations of carbonyl groups at 1734.6 cm^−1^ and stretching vibrations of carboxyl groups at 1629.0 cm^−1^. After complexation, the characteristic bands of tartaric acid and PEIs at 1630.4 cm^−1^ and 1629.0 cm^−1^ were disappeared and replaced by two new bands around 1450–1560 cm^−1^ (belonging to the stretching vibrations of carboxylate), indicating the connection of PEIs with L-tartaric acid. After silica deposition, PEIs@TA-CMS were successfully build up evidenced by characteristic absorption of MSNs, including the antisymmetric stretching vibration of Si–OH at 3452.1 cm^−1^, antisymmetric stretching vibration of Si–O–Si at 1080.3 cm^−1^, symmetric stretching vibration of Si–O–Si at 801.9 cm^−1^, and the bending vibration of Si–O–Si at 464.0 cm^−1^ (Figure 1(B)) (Li et al., 2016; Li et al., 2018).

### 3.2. Characteristics of PEIs@TA-CMS

**Figure 2.**
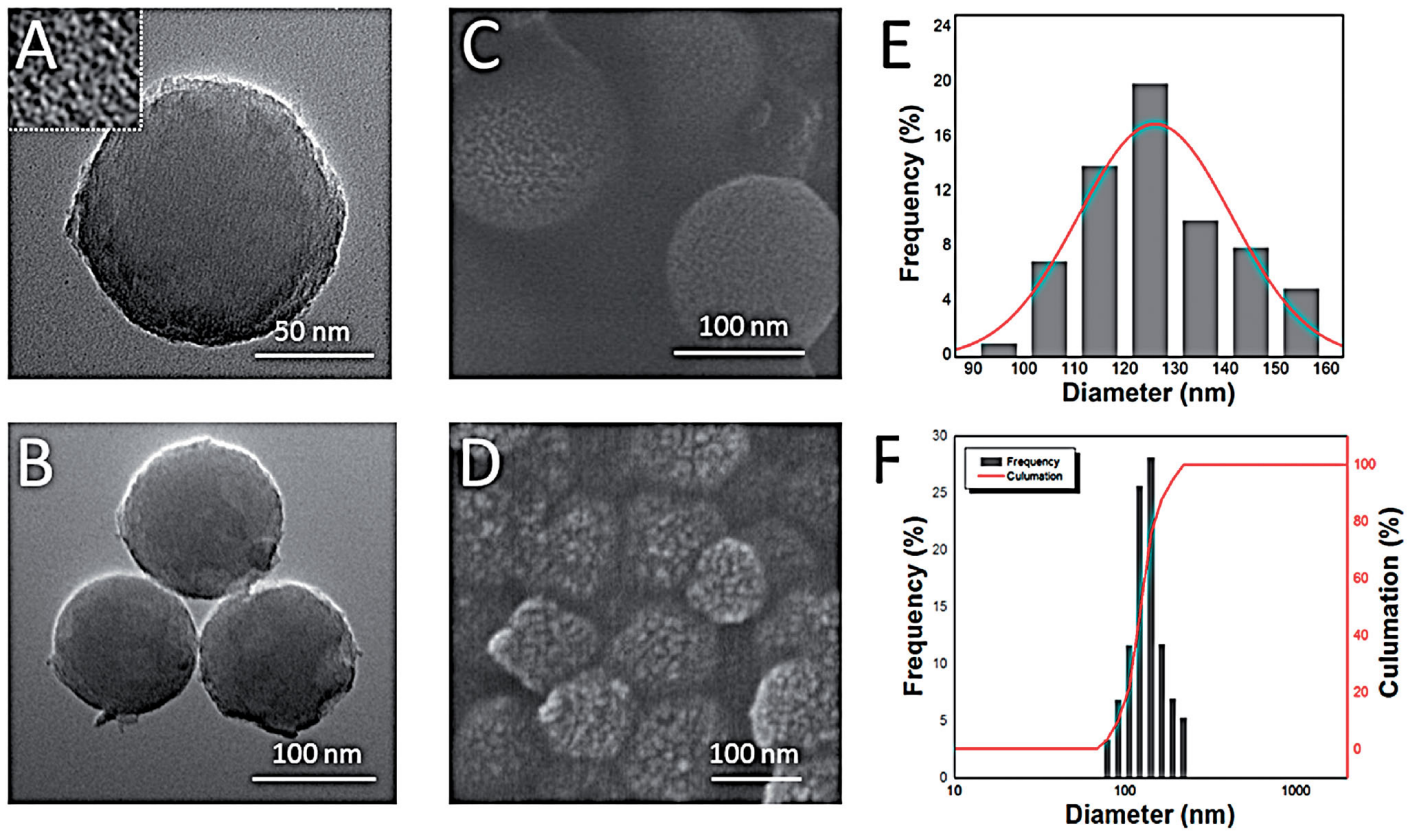
Structural characteristics of PEIs@TA-CMS. (A) and (B) TEM images of PEIs@TA-CMS; (C) and (D) SEM images of PEIs@TA-CMS; (E) particle size distribution of PEIs@TA-CMS calculated from the SEM images; (F) particle size distribution of PEIs@TA-CMS obtained from the Zeta-Potential/Particle Sizer.

By using the novel, easy, low cost chiral silica constructing program, PEIs@TA-CMS was successfully synthesized. We first examined the morphology and pore structure of PEIs@TA-CMS by TEM and SEM. As shown in Figure 2, PEIs@TA-CMS was well-formed spherical nanoparticles with a uniformed diameter of 120–130 nm (calculated from 200 nanoparticles). The nanospheres was monodispersed and exhibited smooth boundary. A large number of slender curved mesochannels were homogeneous distributed on the surface of the PEIs@TA-CMS. The results demonstrated that structurally controlled chiral silica was prepared through the dynamic behavior of the chiral nanocrystalline complex.

**Figure 3.**
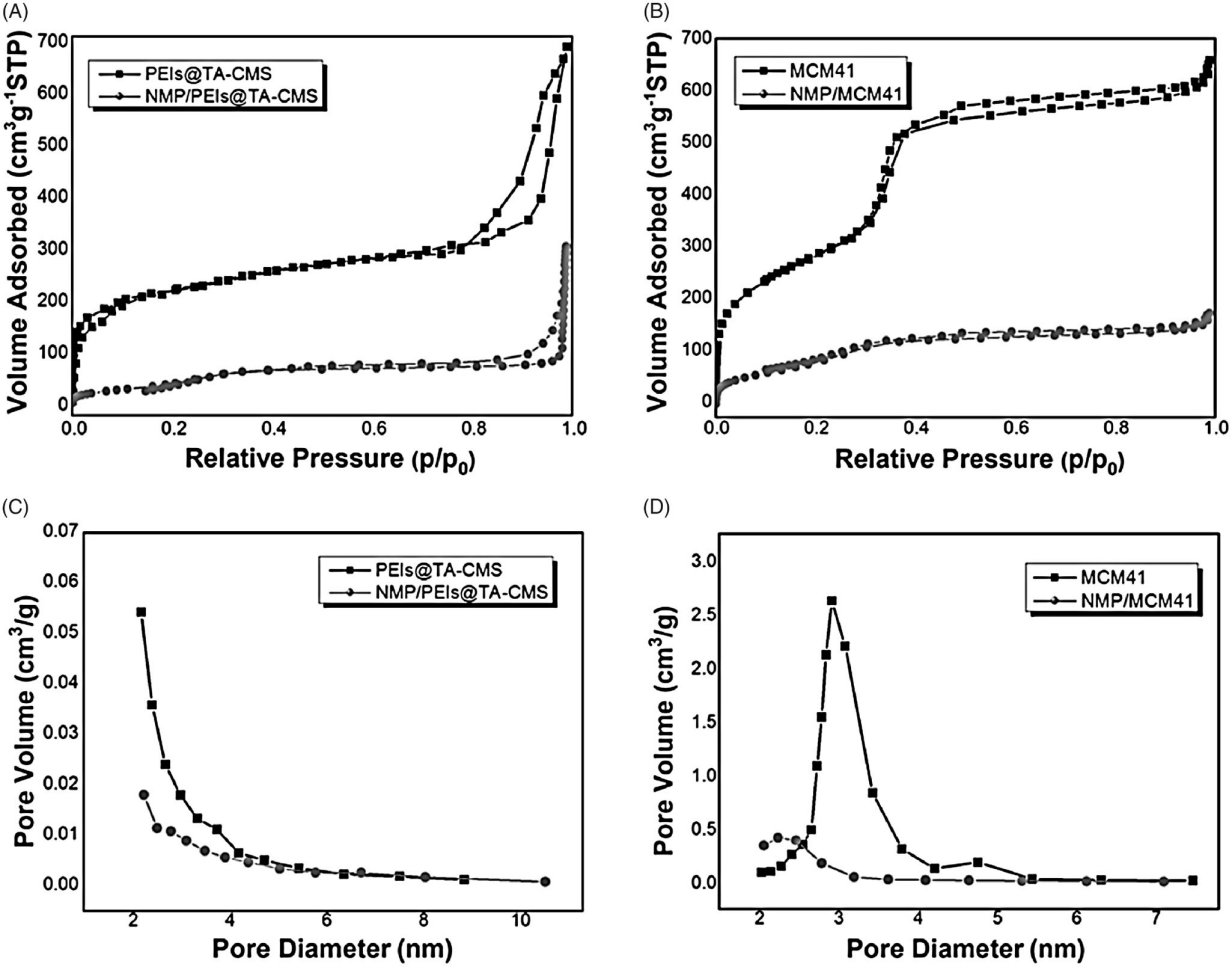
(A) N_2_ adsorption/desorption isotherm of PEIs@TA-CMS and NMP/PEIs@TA-CMS; (B) N_2_ adsorption/desorption isotherm of MCM41 and NMP/MCM41; (C) pore size distribution of PEIs@TA-CMS and NMP/PEIs@TA-CMS; (D) pore size distribution of MCM41 and NMP/MCM41.

The N_2_ nitrogen adsorption–desorption isotherms and pore size distribution curves of PEIs@TA-CMS are respectively displayed in Figure 3(A,C). The isotherm of PEIs@TA-CMS was representative type IV curves with a pronounced capillary condensation step, implying the existence of uniform pores (Li et al., 2018). The S_BET_ of PEIs@TA-CMS was as high as 1009.94 m^2^/g. The pore size distribution curve of PEIs@TA-CMS suggested that nanoparticles had uniformed mesopores, and the average pore size calculated by BJH method was found to be less than 2.21 nm. The texture properties of PEIs@TA-CMS made it a potential candidate for the application as a host platform in a drug delivery system.

**Figure 4.**
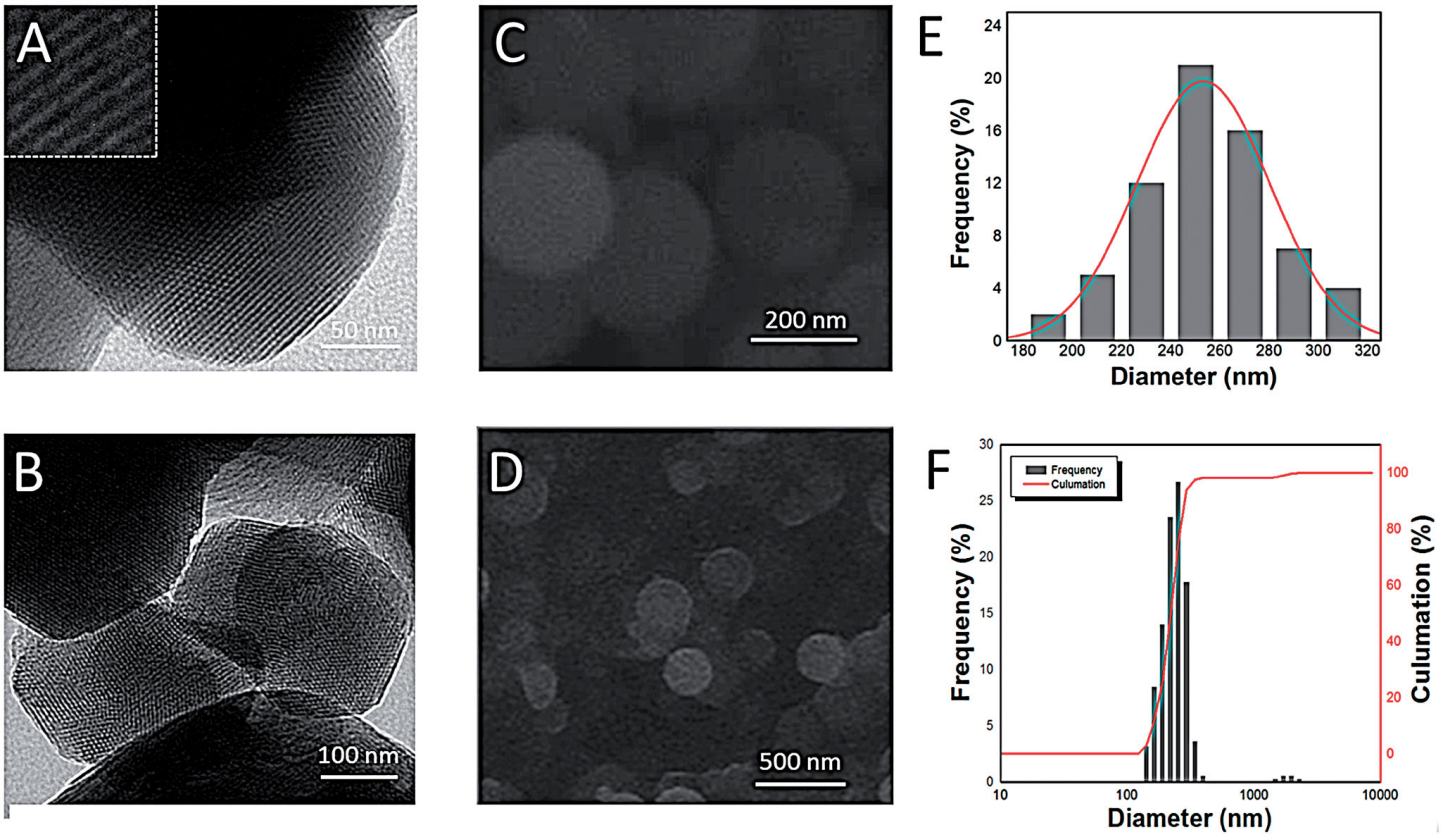
Structural characteristics of MCM41. (A) and (B) TEM images of MCM41; (C) and (D) SEM images of MCM41; (E) particle size distribution of MCM41 calculated from the SEM images; (F) particle size distribution of MCM41 obtained from the Zeta-Potential/Particle Sizer.

MCM41 (a kind of MSN that is widely used in biomedical fields) was also employed as drug carrier and regard as a reference. It was well-formed spherical nanoparticles with a diameter of 240–260 nm (see Figure 4). As displayed in Figure 3(B), the nitrogen adsorption–desorption isotherm of the MCM41 showed type IV patterns with distinct hysteresis loops, implying the presence of uniform mesoporous (with average pore diameter of 3.09 nm, Figure 3(D); Table 1). Additionally, MCM41 had well-developed pore structure, and the BET surface area was found to be 995.33 m^2^/g (Table 1).

**Table 1.** Texture properties of PEIs@TA-CMS and MCM41 before and after drug loading, and the drug loading content.

Sample	S_BET_ (m^2^/g)	V_t_ (cm^3^/g)	W_BJH_ (nm)	Drug loading content (%)
PEIs@TA-CMS	1009.94	0.63	<2.21	/
NMP/PEIs@TA-CMS (1:1)	421.31	0.34	<2.21	52.1% ± 2.62%
NMP/PEIs@TA-CMS (1:2)	304.98	0.24	<2.21	34.5% ± 2.11%
NMP/PEIs@TA-CMS	204.81	0.17	<2.21	24.9% ± 0.67%
MCM41	995.33	0.94	3.09	/
NMP/MCM41	297.14	0.27	2.24	22.9% ± 1.13%

### 3.3. Drug loading amount

As a therapeutic agent for the treatment of blood circulatory disorders in the brain, NMP has been linked to a very low bioavailability (lower than 13%), mostly reasoned by the poorly water solubility. To solve this problem, NMP was incorporated into PEIs@TA-CMS by the solvent deposition method. As indicated by the N_2_ nitrogen adsorption–desorption, the texture parameters (including S_BET_, V_t_) of NMP/PEIs@TA-CMS and NMP/MCM41 were decrease sharply to lower values (Figure 3(A,C) and Table 1). It provided sufficient evidence that NMP was effectually loaded into PEIs@TA-CMS, thus taking up a certain amount of space. Moreover, the W_BJH_ was also moved to lower value because drug molecules were occupied in the nanopores.

The drug loading content was determined by UV-vis analysis, and the results were summarized in Table 1. For a series of NMP/PEIs@TA-CMSs samples, the drug loading content varied from 52.1% to 24.9% (w/w). It was expected to find a significantly negative relationship between the proportion of the carrier and the drug loaded content. The result demonstrated that PEIs@TA-CMS had the ability to load drug with high efficiency, and only a very small fraction of drug was lost during drug loading. The loading content of NMP/MCM41 was 22.9%, which was lower than the loading content of NMP/PEIs@TA-CMS at the same drug: carrier ratio. Apart from the large surface area, the high drug-loading efficiency of PEIs@TA-CMS was also related to its well-developed mesostructure that provided sufficient space for the host of drug molecules.

### 3.4. Interactions between drug and carrier

As presented in Figure 1(C,D), the FTIR spectra of NMP showed band at 3300.1 cm^−1^ corresponded to the stretching vibration of the –NH group, band at 1694.9 cm^−1^ relative to the stretching vibration of carbonyl group, band at 1523.2 cm^−1^ refer to the –NO_2_ stretching, and band at 1308.8 cm^−1^ relative to the C–N stretching vibration (Li et al., 2018). MCM41 had a typical FTIR spectra for mesoporous silica materials, consisting of bands at 3431.1 cm^−1^ (Si–OH antisymmetric stretching vibration), 1084.6 cm^−1^ (Si–O–Si antisymmetric stretching vibration), 801.7 cm^−1^ (Si–O–Si antisymmetric stretching vibration) and 465.3 cm^−1^ (Si–O–Si bending vibration) (Li et al., 2016, 2018). The physical mixtures showed the overlap of the spectra of carriers and NMP with the maintenance of some typical NMP patterns (including bands around 3299.4 cm^−1^, 1524.0 cm^−1^ and 1697.8 cm^−1^), suggesting the absence of chemical interactions or non-covalent weak transitions. After drug loading, both NMP/PEIs@TA-CMS and NMP/MCM41 presented characteristic adsorption of the silica carriers with the disappearance of most of the NMP bands, demonstrating the successful loading of NMP into the nanospace in silica carriers.

### 3.5. Drug crystalline state

**Figure 5.**
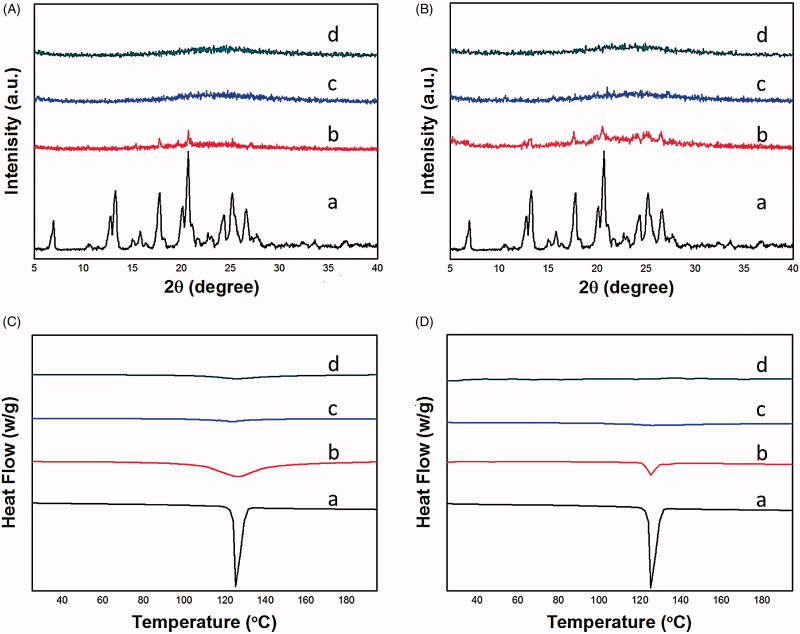
(A) XRD diffractograms of (a) NMP, (b) the physical mixture of NMP and PEIs@TA-CMS, (c) NMP/PEIs@TA-CMS, and (d) PEIs@TA-CMS; (B) XRD diffractograms of (a) NMP, (b) the physical mixture of NMP and MCM41, (c) NMP/MCM41, and (d) MCM41; (C) DSC thermograms of (a) NMP, (b) the physical mixture of NMP and PEIs@TA-CMS, (c) NMP/PEIs@TA-CMS, and (d) PEIs@TA-CMS; (D) DSC thermograms of (a) NMP, (b) the physical mixture of NMP and MCM41, (c) NMP/MCM41, and (d) MCM41.

The XRD analysis of the drug loaded samples was used to determine the transformation of the crystalline state. As collected in Figure 5(A,B), the X-ray diffraction peaks of NMP was highly crystalline with typical peaks at the 2*θ* range of 15.8°, 17.9°, 20.1°, 20.7°, etc., while PEIs@TA-CMS and MCM41 showed broad bands between the 2*θ* range of 5° and 40°, suggested that they were kinds of amorphous materials. Observably, The XRD patterns exhibited very different diffractograms between drug loaded samples and physical mixtures. When drug molecules were physically mixed with PEIs@TA-CMS or MCM41, the crystallization diffraction peaks of the drug were still remained (including peaks at 17.9°, 20.1°, 20.7°) with reduction of intensity. After completion of the drug loading, the XRD patterns of NMP/PEIs@TA-CMS and NMP/MCM41 showed the absence of distinctive peaks. Instead of existing in the original crystalline state, NMP loaded into PEIs@TA-CMS underwent an amorphization process, because the nanopores of mesoporous materials prevented the drug in the noncrystalline state by space confinement.

The crystalline properties were also evaluated by DSC test, in which the melting point is a standard mean to determine the present of crystals. The characteristic melting peaks of pure NMP were observed at 114.4 °C, which was in accordance with the intrinsic melting point (Fu et al., 2012; Liu et al., 2016). In contrast, the DSC curves of PEIs@TA-CMS and MCM41 (Figure 5C,D) were almost smooth lines, meant the amorphous state of silica materials. After being incorporated into PEIs@TA-CMS, NMP/PEIs@TA-CMS showed the amorphous broad peak without any characteristic endothermic event due to the finite-size effect. Similar situation was happened in the case of NMP/MCM41, and no melting peak of NMP was observed. On the contrary, the major melting peaks of NMP were still detectable in the DSC curve of physical mixtures. The DSC results were in good agreements with the XRD findings, and further illustrated the change of drug crystalline state caused by the limited space of mesoporous. It was concluded that PEIs@TA-CMS could be responsible for the amorphization of NMP since the uniformed mesopores exerted pronounced space confinement, which could effectively retain the crystallization of NMP.

### 3.6. *In vitro* drug release study

**Figure 6.**
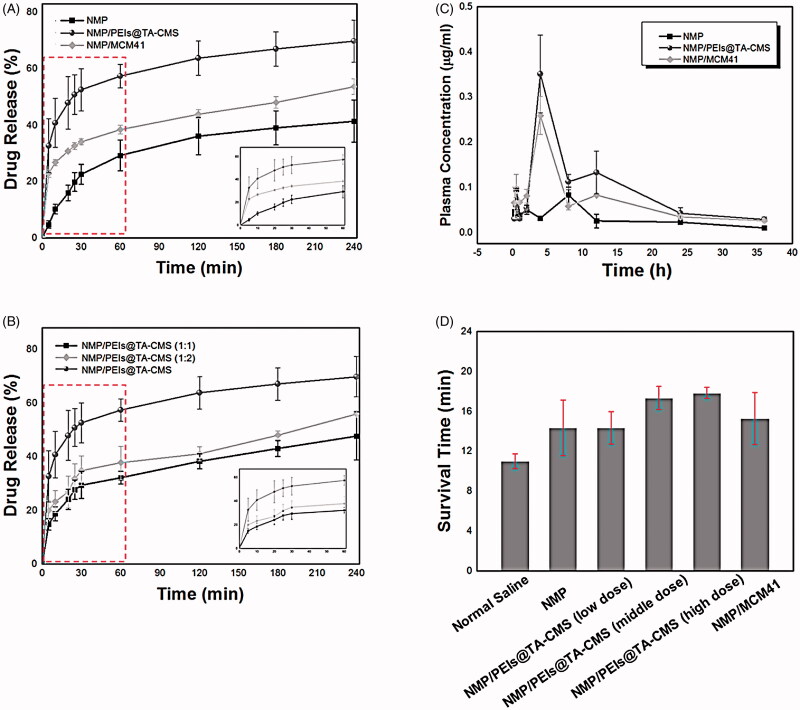
(A) *In vitro* drug release profiles of NMP, NMP/PEIs@TA-CMS and NMP/MCM41; (B) *in vitro* drug release profiles of NMP/PEIs@TA-CMS (1:1), NMP/PEIs@TA-CMS (1:2) and NMP/PEIs@TA-CMS; (C) plasma concentration–time profiles of NMP, NMP/PEIs@TA-CMS and NMP/MCM41; (D) the survival time of mice on cerebral anoxia induced by NaNO_2_. *p < 0.05, ***p < 0.001.

Figure 6(A) depicted the *in vitro* release behaviors of pure NMP, NMP/PEIs@TA-CMS and NMP/MCM41 in 0.5% SDS solution. Pure NMP exhibited slow and incomplete release, in which 29.24% was dissolved within 60 min and 41.32% was dissolved within 240 min. The dissolution rate of NMP was significantly increased after loaded into PEIs@TA-CMS and MCM41 (both at the drug: carrier ratio of 1:3, w/w). NMP/PEIs@TA-CMS could release 70.03% of NMP within 240 min, while NMP/MCM41 could release 53.6% within 240 min. To be specific, the drug release amount of NMP/PEIs@TA-CMS and NMP/MCM41 reached 57.31% and 38.42% within 60 min, which was respectively 1.83-fold higher and 1.31-fold higher than pure NMP. The improvement in drug release was the combined effect of at least two factors: the limitation of drug size and the amorphization of drug molecules (Zhang et al., 2018). NMP crystal could be significant suppression by PEIs@TA-CMS, thus leading to the decrease of particle size. According to Noyes-Whitney equation, a reduction in particle size would result in an increase of effective surface area in diffusion layer, and an improvement in the drug dissolution rate (Lindberg & Lundstedt, 1994; Fu et al., 2012). On the other hand, the amorphization of NMP (as confirmed by the DSC and XRD results) in the limited nanospace was beneficial for the dissolve poorly water-soluble drugs because of a lower thermodynamic barrier to dissolution. The high internal energy and specific volume of the amorphous state relative to the crystalline state also led to enhanced dissolution and bioavailability (Hancock & Zografi, 1997).

It should be noticed that, PEIs@TA-CMS possessed better dissolution-promoting effect then MCM41. It was generally accepted that, drug release from mesoporous silica matrix could be regarded as a diffusion-controlled process mainly dominated by the pore size and pore structure (Zhang et al., 2015). However, in this case, MCM41 with the larger pore size released much smaller than PEIs@TA-CMS with smaller pore size (2.8 nm), which was exactly the opposite of the previous studies (Perez et al., 2017). Apart from the pore diameter, the pore geometry also had great influence on the drug release behavior of mesoporous silica. PEIs@TA-CMS possessed curved short nanopores with good connectivity (as displayed in the embedded picture in Figure 2(A)), and could reduce the diffusion hindrance and facilitated the mass transfer into the bulk solution while preforming the dissolution task. Whereas the lattice fringes pore structure of MCM41 (the embedded picture in Figure 4(A)) limited the accessibility of pores, thus resulting in higher diffusion resistance and longer diffusion distance (Wang et al., 2014; Hu et al., 2012).

The enhancement of NMP dissolution was also largely related to the drug: carrier ratio, in which the release rate was positively correlated with the proportion of the carrier. As indicated in Figure 6(B), the series of NMP/PEIs@TA-CMS formulations showed enhanced release in both release rate and accumulative release percentage compared with pure NMP. The cumulative drug release amount of NMP/PEIs@TA-CMS was in the range of 47.67% to 70.03% within 240 min, and was followed the order: NMP/PEIs@TA-CMS (1:1) sample < NMP/2PEIs@TA-CMS (1:2) < NMP/3PEIs@TA-CMS sample. It seemed that, the release profile of NMP can be effectively regulated by mixing with different carrier ratios.

### 3.7. Pharmacodynamics study

To evaluate the performance of PEIs@TA-CMS on promoting the drug absorption, *in vivo* pharmacokinetics study was conducted. The plasma concentration time profiles of pure NMP, NMP/PEIs@TA-CMS and NMP/MCM41 after oral administration are displayed in Figure 6(C), and the main pharmacokinetic parameters are summarized in Table 2. In the plasma concentration time profiles of pure NMP, insufficient and poor absorption was observed, with the maximum concentration (*C*_max_) of 0.10 μg/mL. Double peaks appeared at 0.5 h (*T*_max_) and 8 h post administration, strongly suggested the existence of enterohepatic circulation. Compared to pure NMP, NMP/MCM41 and NMP/PEIs@TA-CMS showed clear differences in the shape of the curves, in which only one main peak was found in each curve, and the *T*_max_ reached later. For both drug loaded samples, the plasma concentration of NMP gradually increased up to the maximum concentration at 4 h (*T*_max_), and then the value started to decline. The *C*_max_ values were raised to 0.35 μg/mL and 0.26 μg/mL for NMP/PEIs@TA-CMS and NMP/MCM41, which were 3.68-fold and 2.72-fold higher than that of pure NMP, respectively. Furthermore, as shown in Table 2, the relative bioavailability of pure NMP, NMP/MCM41 and NMP/PEIs@TA-CMS were 100%, 270.53% and 326.20%, respectively. Undoubtedly, both MCM41 and PEIs@TA-CMS could significantly improve the oral adsorption of NMP. On one hand, the dissolution process could be regard as the rate-limiting step for the oral absorption of poorly water-soluble drugs. By loading into the carries, the mesopores effectively restricted NMP in an amorphous state within the channels, which facilitated the drug dissolution. The increased drug dissolution caused by the transformation of the crystalline state could lead to the improvement of absorption after oral dosing (Fu et al., 2012; Liu et al., 2016). On the other hand, MSNs were carriers with unique structural and textural features. The high surface area made them easily adhering on the small intestine wall, thus favored the absorption through gastrointestinal. Meanwhile, the inorganic “rigid” matrices with thermal/chemical stability provided better control for drug delivery *in vivo*.

**Table 2.** Pharmacokinetic parameters obtained after oral administration of NMP, NMP/PEIs@TA-CMS and NMP/MCM41.

Pharmacokinetic parameter	NMP	NMP/PEIs@TA-CMS	NMP/MCM41
AUC_0-∞_ (mg/L*h)	1.19 ± 0.16	3.89 ± 0.33	3.29 ± 0.21
MRT_0-∞_ (h)	8.84 ± 1.73	9.48 ± 2.80	14.00 ± 1.62
*t*_1/2_ (h)	10.87 ± 1.13	12.64 ± 2.57	18.65 ± 0.70
*T*_max_ (h)	0.5 ± 0	4 ± 0	4 ± 0
*C*_max_ (mg/L)	0.10 ± 0.02	0.35 ± 0.09	0.26 ± 0.04
Relative bioavailability (%)	100%	326.20%	270.53%

Among them, the PEIs@TA-CMS presented beneficial oral absorption with higher bioavailability and higher maximum drug concentration (which was 1.20-fold higher in AUC, and 1.45-fold higher in *C*_max_ then NMP/MCM41, Table 2), which was in accordance with the *in vitro* release results. *In vivo* pharmacodynamics study illustrated that PEIs@TA-CMS exhibited great potential for release and delivery of NMP.

### 3.8. Pharmacodynamics study

As a dihydropyridine calcium channel blocker, NMP was globally used to treat resultant ischemia and cerebral vasospasm. To further probe the function of NMP/PEIs@TA-CMS and NMP/MCM41, pharmacodynamics study was carried out. In this experiment, NMP, NMP/PEIs@TA-CMS (middle dose) and NMP/MCM41 were administered orally to animals at the dose of 0.5 mg; 0.4 mL NaNO_2_ (w/v) was intraperitoneal injected to induce cerebral anoxia. As indicated in Figure 6(D), in Normal Saline group which served as negative control, the survival time of mice after the injection of NaNO_2_ was found to be 10.98 min, suggesting the successfully establishment of mice cerebral anoxia model. In NMP group, the administration of NMP motivated the oxygen utilization coefficient and enabled the oxygen supply in blood, and the survival time of mice was prolonged to 14.33 min. Besides, owing to the enhanced bioavailability, the survival period of NMP/PEIs@TA-CMS (middle dose) and NMP/MCM41 underwent a further prolongation to 17.33 min and 15.26 min. Compared to NMP and NMP/MCM41, NMP/PEIs@TA-CMS was found to be more powerful in treatment of cerebral anoxia under the same dose. The order of survival time was NMP/PEIs@TA-CMS (middle dose) > NMP/MCM41 > pure NMP, which was in accord with the order of bioavailability. Moreover, with the aim of exploring the effect of dose, mice in NMP/PEIs@TA-CMS (low dose) group and NMP/PEIs@TA-CMS (high dose) group were orally administered half and twice of the dose in NMP/PEIs@TA-CMS (middle dose) group, respectively. The results revealed that NMP/PEIs@TA-CMS also significantly and dose-dependently postponed the coming of death to 14.34 min and 18.83 min, respectively. All these results provided adequate evidence and convincing confirmed the superior of PEIs@TA-CMS in improving the oral adsorption of poorly water-soluble.

## 4. Conclusion

The present study reported a novel drug delivery system established by PEIs@TA-CMS with chiral structure and sphere appearance. For the first time, chiral mesoporous silica nanoparticle named PEIs@TA-CMS was developed through a facile and controllable strategy by using a chiral crystalline complex as templates, scaffolds and catalysts. PEIs@TA-CMS was well-formed spherical nanoparticles in a uniformed diameter of 120–130 nm with curved nanopores. Then crystal NMP was effectively loaded into PEIs@TA-CMS and transformed to an amorphous state due to the space confinement. After that, both the drug release rate and amount was significantly improved, and best result came from NMP/PEIs@TA-CMS sample (at drug: carrier weight ratio of 1:3), in which 70.03% of NMP could release within 240 min. Notably, PEIs@TA-CMS was a drug delivery platform to realize the efficiency delivery of NMP with satisfactory relative bioavailability (up to 326.2%) and pharmacodynamics effects. Compared to MCM41, it had superiority in improving the oral adsorption of poorly water-soluble drug to a satisfactory level. Undoubtedly this study will be of great significant in the rational and flexible design of drug carriers.
